# Psychometric properties of the Arabic Fear of Happiness Scale in a sample of Lebanese adults

**DOI:** 10.1371/journal.pone.0356389

**Published:** 2026-09-15

**Authors:** Marie Anne El Khoury, Diana Malaeb, Fouad Sakr, Mariam Dabbous, Feten Fekih-Romdhane, Souheil Hallit, Sahar Obeid

**Affiliations:** 1 Department of Psychology and Education, School of Arts and Sciences, Lebanese American University, Beirut, Lebanon; 2 College of Pharmacy, Gulf Medical University, Ajman, United Arab Emirates; 3 School of Pharmacy, Lebanese International University, Beirut, Lebanon; 4 The Tunisian Center of Early Intervention in Psychosis, Department of Psychiatry “Ibn Omrane”, Razi Hospital, Manouba Tunisia; 5 Faculty of Medicine of Tunis, Tunis El Manar University, Tunis, Tunisia; 6 School of Medicine and Medical Sciences, Holy Spirit University of Kaslik, Jounieh, Lebanon; 7 Applied Science Research Center, Applied Science Private University, Amman, Jordan; 8 Department of Psychology and Education, School of Arts and Sciences, Lebanese American University, Jbeil, Lebanon; De Montfort University, UNITED KINGDOM OF GREAT BRITAIN AND NORTHERN IRELAND

## Abstract

**Background:**

Despite the prominence of happiness as a psychological ideal, not all individuals experience it as inherently positive. In some cultural and psychological contexts, happiness may be approached with ambivalence or apprehension, reflecting concerns about its potential consequences. The Fear of Happiness Scale (FHS) was developed to assess such beliefs. This study evaluates the psychometric properties of the Arabic version of the FHS among Lebanese adults.

**Methods:**

The FHS was translated to Arabic following a conventional forward-backward translation procedure and was administered to a sample of 484 Lebanese adults along with the Patient Health Questionnaire, the WHO-5 Well-Being Index, and the Generalized Anxiety Disorder scale for convergent validity. The factor structure was studied by Confirmatory Factor Analysis with weighted least squares mean and variance adjusted estimation.

**Results:**

Results suggested an acceptable fit of the one-factor model of the FHS scale with good internal consistency (ω = 0.95 / α = 0.95) and excellent convergent validity as shown by the Average Variance Extracted (AVE = 0.87). Measurement invariance was established across sex groups, with no significant difference being reported between males and females in terms of FHS scores. Finally, adequate convergent validity was tested and found to be adequate, with FHS scores found to be correlated negatively with wellbeing and positively with depression and anxiety.

**Conclusion:**

This study suggests that the Arabic version of the Fear of Happiness Scale is a valuable valid tool for researchers and clinicians working with Arabic-speaking populations. It is anticipated that the Arabic FHS will be advantageous for healthcare professionals and researchers working with Arabic-speaking people around the world.

## Introduction

Happiness is widely regarded as one of the highest values and a core human pursuit [1], more specifically in Western cultures [2]. It’s an important topic in a broad range of psychology research and a central one in positive psychology [3]. Despite this, it remains a loose, complex, and multifaceted construct that encompasses both subjective wellbeing and life satisfaction [4,5]. The most accepted definition is proposed by Diener et al. [6] who described happiness as encompassing three key elements: infrequent occurrences of negative emotions, frequent experiences of positive emotions, and a high degree of life satisfaction. Studies indicate that experiencing happiness can aid in achieving significant goals, help with attention span, and help individuals become more receptive to new stimuli by increasing attentional flexibility [7]. Moreover, experiencing more intense positive emotional states has been associated with more favourable physical health which may, in turn, support overall psychological functioning [8].

### Cultural differences

Despite extensive research, happiness and subjective wellbeing are frequently approached in ways that overlook their conceptual depth and contextual complexity. Within positive psychology, happiness is often assumed to hold a universally central place in human values [9,10]. Whereas across many cultural settings, values other than happiness are prioritized, and in certain contexts happiness itself may be approached with caution or viewed unfavorably [11].

Intercultural studies revealed that views on happiness vary depending on whether a culture leans more towards collectivism or individualism. Cultural contexts that emphasize individualism are often associated with higher reported levels of life satisfaction compared to more collectivist settings. This pattern is commonly attributed to the value placed on personal autonomy, self-determination, and the active cultivation of positive emotional experiences [12]. Therefore, with the rise of positive psychology and the increasing amount of research on happiness and wellbeing, interestingly a less well-known and counterintuitive construct appeared, the fear of happiness defined as the belief that experiencing happiness may lead to negative consequences. Although the notion of aversion to happiness existed earlier under the term of cherophobia [13], the construct was systematically conceptualized and operationalized by Joshanloo [11]. Fear of happiness reflects beliefs that joy is transient, that positive emotions are often followed by misfortune, or that happiness may attract envy, bad luck, or moral decline.

Cultural studies suggest that fear of happiness is particularly salient in societies where beliefs about fate, envy, and moral restraint shape emotional experiences [11]. This stems from various reasons like the notion that joy is ephemeral and ultimately replaced by sorrow [11]. Some people might worry that their happiness might make them targets for envy or evil eye [14]. Religious belief systems may also contribute to fear of happiness, particularly when they emphasize caution toward worldly pleasures that are seen as morally compromising or spiritually risky [11]. Indeed, looking into different studies from Eastern cultures we can find that in Turkey, there’s a belief that excessive joy invites bad luck [15,16]. Taoist philosophy, prevalent in countries such as Japan and China, emphasizes change and the cyclical nature of existence. This philosophy questions the enduring value of happiness, given its inevitable transition to unhappiness [17]. In the United Arab Emirates, a study showed that university students often define happiness in collective terms, such as familial pride or religious fulfillment [18].

### Fear of Happiness Scale

Two scales tried to address the fear of happiness aiming to measure people’s aversion to happiness. The first one was proposed by Gilbert [19], and is a 9-item scale that assesses individuals’ beliefs and anxieties about the potential downsides of experiencing happiness and positive emotions. This scale comprised two factors later reduced to one. In its Iranian version, they also found two factors: “fear of experience of happiness” and “fear of the consequences of happiness” [20]. The second and most used scale to assess the fear of happiness was developed by Joshanloo [11], the Fear of Happiness Scale (FHS). The scale consists of 5 items, organized into a single factor. Each item is rated on a 7-point Likert scale (1 = Strongly disagree, 7 = Strongly agree). The total score reflects the degree to which an individual holds a belief that happiness is followed by negative consequences. Each item directly assesses a core belief about the dangers of happiness, making the scale highly specific in capturing this psychological construct. It has shown reliability and validity across various cultures and has been examined in multiple countries, including New Zealand, Iran, Singapore, Hong Kong, Malaysia, Japan, Korea, Taiwan, India, Russia, Brazil, Pakistan, and Kuwait [21], Turkey [22], Portugal [23], and Poland [12]. The scale was confirmed to measure a single concept and demonstrated good internal consistency (Cronbach’s alpha ranging from 0.70 to 0.87) across 16 countries [12,21–23] with the exception of India where the model fit didn’t reach an acceptable level [21].

Though there is no difference in fear of happiness among genders, a study found that women’s fear of happiness is linked to experiences of depersonalization, childhood emotional neglect, and physical abuse while men’s fear of happiness seems to be connected to their tendency to become deeply absorbed in activities [24]. Absorption reflects a tendency toward intense immersion in internal experiences or activities and has been conceptualized as a dissociative-like cognitive style. This pattern suggests that, in men, fear of happiness may be more closely tied to cognitive engagement and experiential immersion rather than to trauma related psychological consequences, highlighting potential gender-specific pathways underlying fear of happiness. Additionally, research indicates that fear of happiness is negatively linked to life satisfaction, wellbeing, and gratitude while positively related to depression [25], anxiety [19,26] and cultural factors like conformity and cynicism [21,27]. Lastly, a study found a positive relation between fear of happiness and insecure attachment [28].

### The Lebanese context

Lebanon, while rooted in the Middle East, has a strong Western influence due to its geography, history and globalization, with its population significantly impacted by Western ideas, thus offering a unique cultural landscape, add to this major life stressors that occurred in recent years. Studies on happiness and positivity in Lebanon showed that Lebanese citizens suffer from widespread dissatisfaction with their country, with 63% reporting it hinders their personal growth [29]. Research conducted among Lebanese young adults suggests that positivity is shaped by demographic and contextual factors, including age, gender, and geographic location, which in turn affects their overall life outcomes [30]. Within the Lebanese population, happiness has been found to be associated with both physical health and religiosity, though men reported higher levels of happiness, health, and religiosity than women, with these factors being interconnected [31]. These patterns point to the central role of religion in Lebanese society and its close association with how happiness is perceived and experienced. On the other hand, in Lebanon, there’s a common popular belief in the evil eye [32,33], add to this that the Lebanese culture tend to show a mix between collectivism and individualism [34,35], thus all factors that are listed as contributors to the fear of happiness exist in Lebanon making this an interesting topic to assess in the Lebanese population.

### Study objectives

To our knowledge, no psychometric validation of an Arabic-language version of the FHS has been published to this day, although cross-cultural studies have included participants from Arabic-speaking countries using non-Arabic versions of the scale. Therefore, this research aimed to explore the validity of an Arabic version of the FHS in a Lebanese sample and evaluate its psychometric properties. Specifically, we sought to: (1) evaluate the factorial structure of the Arabic FHS; (2) assess its internal consistency and measurement invariance across sex; and (3) examine its concurrent validity by investigating associations with well-being and psychological distress (depression, anxiety).

## Methods

### Ethics approval and consent to participate

Ethics approval for this study was obtained from the ethics committee of the School of Pharmacy at the Lebanese International University (2024ERC-024-LIUSOP). Written informed consent was obtained from all subjects; the online submission of the soft copy was considered equivalent to receiving a written informed consent. All methods were performed in accordance with the relevant guidelines and regulations.

### Study design and participants

A total of 484 Lebanese participants were enrolled in this cross-sectional study that was conducted between March and April 2024 in several Lebanese governorates. The research team employed a snowball sampling method to collect data. This sampling approach allowed to increase participation rates by leveraging existing social networks. It also ensured diversity in age, geographic location and socioeconomic background. Included were persons of Lebanese origin, residing in Lebanon, and aged 18 years and above. Excluded were people not fulfilling those criteria and those who did not complete the questionnaire. Participants were initially approached in person and invited to complete an online survey via Google Forms. Those who agreed were asked to share the survey link with their acquaintances. Participants received an online link to the study. Upon clicking the link, they were presented with an informed consent document outlining the study’s purpose, anonymity, and voluntary nature. To encourage honest responses, participants were assured of confidentiality and provided with clear instructions about the survey’s goals. No incentives were offered for participation.

The survey was administered using Google Forms with the option “limit to one response” enabled, which required participants to access the form through a Google account and prevented multiple submissions from the same account. The platform did not provide access to IP addresses or geolocation data. Response time was not recorded. Due to the use of online snowball sampling, the response rate could not be calculated.

### Minimal sample size

Following Mundfrom et al. (2005) [36], sample size adequacy for confirmatory factor analysis was assessed using participant-to-item ratio guidelines, with suggested ratios ranging from 3 to 20 participants per item. With five items in the FHS, the final sample of 484 participants clearly exceeds these recommendations [36] ensuring adequate statistical power.

### Translation procedure

**Fig 1:**
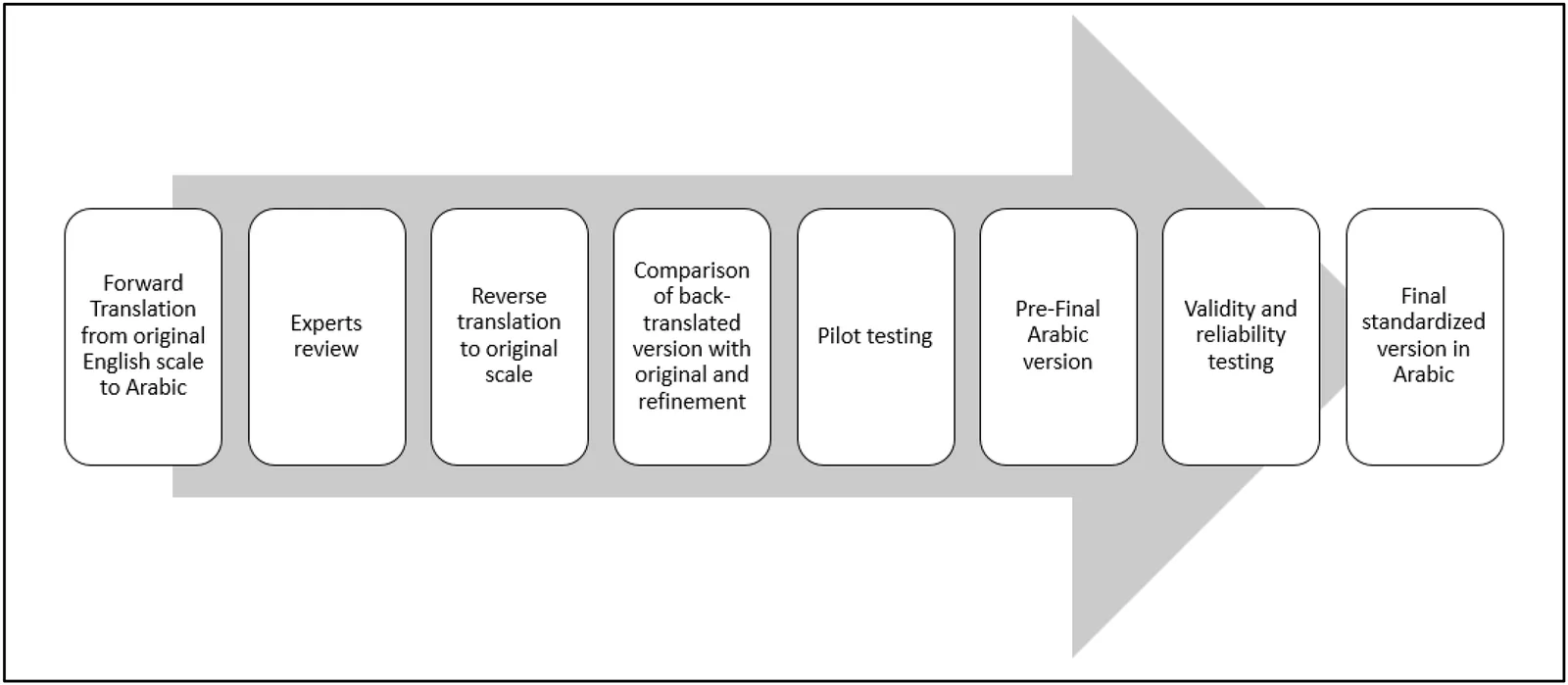
Translation and Adaptation Process of the Arabic FHS Scale.

The Arabic version of the FHS scale was carefully translated and culturally adapted. A rigorous process involving forward and back-translation (Fig 1), along with expert review by bilingual professionals, ensured semantic and conceptual equivalence between the original and Arabic versions. Translation was in line with international standards [37]. The FHS was independently translated from English to Arabic by two bilingual experts in psychology and psychometrics. The translators focused on maintaining conceptual accuracy rather than literal translation to ensure cultural relevance. A panel of three Arabic-speaking psychologists reviewed the translations. This version was assessed for linguistic clarity, cultural appropriateness and psychological construct equivalence. The reconciled Arabic version was back-translated into English by two independent bilingual translators who had no prior exposure to the original scale. This step ensured that the Arabic version retained the original meaning and that no significant distortions occurred during translation. The back-translated version was then compared to the original English FHS, and minor refinements were made where necessary. A pilot test, done on 30 participants, confirmed the clarity of the translated items before the main study commenced. This step was designed to address any potential misunderstandings concerning the language and readability of the items [38].

### Measures

The questionnaire was administered entirely in Arabic, the native language of Lebanese people. Participants were required to provide informed consent before proceeding with the questionnaire. The survey itself took approximately 10–15 minutes to complete.

1. *Sociodemographic Survey*: Participants provided self-reports on their age, sex, marital status, mental health disorder status, and the Household Crowding Index (HCI), which reflects the socioeconomic status (calculated by dividing the number of persons by that of the rooms in the house besides the kitchen and bathrooms) [39].
2. *The Fear of Happiness Scale (FHS)* is a five-item questionnaire designed to measure the belief that excessive happiness can lead to negative consequences. Participants rate their agreement with each statement on a seven-point scale, ranging from strongly disagree to strongly agree, with a higher score indicating a higher fear of happiness. The items are: (1) “I prefer not to be too joyful, because usually joy is followed by sadness.” (2) “I believe the more cheerful and happy I am, the more I should expect bad things to occur in my life.”(3)“Disasters often follow good fortune.” (4) “Having lots of joy and fun causes bad things to happen.” (5)“Excessive joy has some bad consequences” [11]. We obtained permission from the original author to use and validate the scale in our study.
3. *The World Health Organization – Five Well-being Index (WHO-5)*, is a widely used questionnaire assessing subjective psychological well-being [40]. In the validated Arabic version, the CFA confirmed a unidimensional structure, and McDonald’s Omega (ω = 0.95) indicating excellent internal reliability [41,42]. It is formed of 5 items scored on a 6-point Likert scale with anchors ranging from “at no time” to “all the time” (e.g., *In the last two weeks, I have felt cheerful in good spirits*). Items are summed on a scale of 0–25, with higher scores reflecting higher wellbeing (ω = 0.94 / α = 0.94 in this study).
4. *The Patient Health Questionnaire 9 (PHQ-9)* is a widely used and validated tool for screening patients for depression [43]. Validated in Lebanon [44], the scale is composed of 9 items, evaluating symptoms of depression in the last two weeks (e.g., *Little interest or pleasure in doing things*, *over the last two weeks*). It is rated on a 4-point Likert scale where 0 indicates absence of symptoms and 3 the presence of symptoms nearly every day. Higher scores indicate more severe depression (ω = 0.91 / α = 0.91 in this study).
5. *The Generalized Anxiety Disorder-5 scale (GAD-5)* is a 5-item scale assessing symptoms of anxiety in the last 2 weeks (e.g., *I feel nervous, anxious, or about to burst*) [45]. Its validation in Lebanon confirmed a one-factor model with excellent internal consistency (ω = 0.95; α = 0.95 [46]). CFA results showed acceptable model fit (CFI = 0.989, RMSEA = 0.100, SRMR = 0.014), and measurement invariance was supported across sex, age, and education level. It is rated on a 10-point Likert scale, where 0 indicates “does not describe me” and 10 “describes me exactly.” (ω = .97 / α = .97 in this study).

### Analytic strategy

There were no missing responses in the dataset. We used data from the total sample to conduct a CFA using RStudio, “lavaan” and “SemTools” programs [47,48]. Parameter estimates were obtained using the Mean and Variance (WLSMV) estimation method, the most suitable for ordinal data [49]. The calculated fit indices were the Root Mean Square Error of Approximation (RMSEA ≤ 0.08), the Standardized Root Mean Square Residual (SRMR ≤ 0.05), the Tucker-Lewis Index (TLI) and the Comparative Fit Index (CFI) (≥ 0.90) [50]. Multivariate normality was not verified as shown by the Mardia’s skewness and kurtosis values.

To examine invariance of FHS scores by sex and self-reported presence of mental disorders, we conducted multi-group CFA [51] using the total sample. Measurement invariance was assessed at the configural, metric, scalar and strict levels [52]. We accepted ΔCFI ≤ 0.010 and ΔRMSEA ≤ 0.015 or ΔSRMR ≤ 0.010 as evidence of invariance [53].

Internal reliability was assessed using McDonald’s ω and Cronbach’s α, with values greater than 0.70 reflecting adequate composite reliability [54]. The FHS scores were considered normally distributed following the skewness (= 0.675) and kurtosis (= −0.258) values varying between −1 and +1. Therefore, the independent samples t test was used to compare means between genders, whereas the Pearson correlation test was used to correlate the FHS scores with the other scales.

## Results

Four hundred eighty-four participants completed the survey, with a mean age of 27.74 ± 11.17 years, 68.4% females and a mean HCI of 1.12 ± 0.47. Moreover, 108 (11.9%) of participants self-reported presence of mental disorders.

### Confirmatory factor analysis of the FHS scale

CFA indicated that the fit of the one-factor model of the FHS scale was good: Confirmatory factor analysis (CFA) was conducted to examine the one-factor model of the FHS scale. The model resulted in the following fit indices: RMSEA = 0.112 (90% CI 0.079, 0.148), SRMR = 0.022, CFI = 1.000, TLI = 0.999. The standardized estimates of factor loadings were all adequate (Table 1). Composite reliability of scores was adequate in the total sample (ω = 0.95 / α = 0.95). The convergent validity, as reported by the AVE value, was excellent (AVE = 0.89).

**Table 1:** Standardized loading factors of the Fear of Happiness Scale items in English and Arabic.

Item	Loading factor
1. I prefer not to be too joyful, because usually joy is followed by sadness. . أفضّل ألا أكون فرحًا جدًا، لأن الفرح عادةً ما يتبعه حزن	0.89
2. I believe the more cheerful and happy I am, the more I should expect bad things to occur in my life. . أعتقد أنه كلما كنت مبتهجًا وسعيدًا، كلما توقعت حدوث أشياء سيئة في حياتي	0.96
3. Disasters often follow good fortune. . الكوارث غالبا ما تتبع الحظ السعيد	0.93
4. Having lots of joy and fun causes bad things to happen. . إن الحصول على الكثير من الفرح والمرح يؤدي إلى حدوث أشياء سيئة	0.96
5. Excessive joy has some bad consequences. . الفرح المفرط له بعض العواقب السيئة	0.93

### Sex invariance

We were able to show the invariance by sex and presence of self-reported mental disorders status at all levels (Table 2). No significant difference was found between males (Mean = 13.27; SD = 7.52) and females (Mean = 13.36; SD = 7.04) in terms of FHS scores, t(482) = −0.12, p = 0.904, Cohen’s d = −0.012. However, participants with self-reported mental health disorders had higher mean FHS scores compared to those who do not (Mean = 13.10, SD = 7.13 vs Mean = 16.45; SD = 7.36; t(482) = −2.60, p = 0.010, Cohen’s d = 0.469).

**Table 2:** Measurement Invariance of the Fear of Happiness Scale in the total sample.

Model	CFI	RMSEA	SRMR	Model Comparison	ΔCFI	ΔRMSEA	ΔSRMR
**Model 1: Invariance by sex**							
**Configural**	1.000	0.093	0.020				
**Metric**	1.000	0.101	0.023	Configural vs metric	<0.001	0.008	0.003
**Scalar**	1.000	0.020	0.020	Metric vs scalar	<0.001	0.082	0.003
**Strict**	1.000	0.062	0.026	Scalar vs strict	<0.001	0.042	0.006
**Model 2: Invariance by self-reported mental disorders status**							
**Configural**	0.999	0.125	0.021				
**Metric**	0.999	0.118	0.023	Configural vs metric	<0.001	0.007	0.002
**Scalar**	1.000	0.066	0.021	Metric vs scalar	0.001	0.052	0.002

*Note.* CFI = Comparative fit index; RMSEA = root mean square error of approximation; SRMR = Standardized root mean square residual. Strict invariance was not calculated in Model 2 due to the small size of the group with self-reported mental disorders and the ordinal nature of the indicators.

### Concurrent validity

Higher FHS scores were significantly associated with higher depression, higher anxiety and lower wellbeing (Table 3).

**Table 3:** Correlation matrix.

	Mean ± SD	1	2	3
**1. Fear of happiness**	13.33 ± 7.19	1		
**2. Depression**	9.09 ± 6.43	0.34***	1	
**3. Anxiety**	18.62 ± 12.15	0.33***	0.43***	1
**4. Wellbeing**	15.48 ± 5.58	−0.25***	−0.49***	−0.44***

***p < 0.001.

## Discussion

This study aimed to evaluate the Arabic version of the FHS in a sample of Arabic-speaking adults. Our first hypothesis was tested using CFA to evaluate the scale’s structural validity. Results partially supported the original one-factor structure of the scale; the CFA indicated excellent incremental fit and residual fit (SRMR), whereas the RMSEA value exceeded the conventional cutoff.

These results align with previous research done by Joshanloo et al. [21] on 14 countries, which also reported excellent fit indices except for the RMSEA. Other validation studies reported similar results, such as Turkey [22], Portugal [23] and Poland [12]. Specifically, in models characterized by low degrees of freedom (df < 50), the RMSEA is frequently susceptible to upward bias [55]. Considering that our tested model possessed limited degrees of freedom (df = 5), the RMSEA may overestimate model misfit and therefore lack the precision necessary for an accurate fit assessment. Consequently, we prioritized more resilient metrics such as CFI and SRMR [56]. The excellent CFI and RMSEA provide evidence supporting the adequacy of the one-factor model despite the inflated RMSEA. The results suggest that the Arabic FHS captures a largely unidimensional construct, while underscoring the importance of continuous examination of its structural properties across populations and contexts.

Additionally, our second hypothesis is confirmed, reliability was found good and validity excellent (ω = 0.95 / α = 0.95), aligning with all previous studies [12,21–23]. Validations of different translations are aligned with our results: Turkey ω = 0.86; [22], Portugal ω = 0.94 [23], and Poland ω = 0.85 [12]. These findings indicate that the scale items are highly coherent and reliably measure fear of happiness within the present sample.

Furthermore, measurement invariance testing confirmed configural, metric and scalar invariance across sex, consistent with other research [12,21–24]. This suggests that the factor structure, item loadings and responses remain stable across male and female participants, reflecting its applicability and validity in diverse populations.

As per our third hypothesis, concurrent validity was met. Fear of happiness appears to be accompanied by broader psychological vulnerability, including higher levels of depression and anxiety and lower overall well-being. Consistent with this finding, previous research has linked fear of happiness to reduced subjective well-being [21], positive affect, life satisfaction and subjective happiness [16,22]. In contrast, fear of happiness has been shown to positively correlate with negative affect [22], depression [25] along with verticality, conformity, societal cynicism, and dynamic externality [21]. Correlations were in the expected direction, though of moderate strength. The magnitude of these associations is comparable to what has been reported in prior work. For example in an adolescent sample, fear of happiness showed a moderate correlation with depressive symptoms (r = 0.45) [57]. Cross-cultural work has also reported small-to-moderate negative association between fear of happiness and life satisfaction [21], consistent with the current pattern of modest effects. This pattern is theoretically coherent.

Fear of happiness represents a belief system regarding the potential consequences of positive emotions rather than a direct measure of psychological distress, which may account for the moderate strength of associations. Moreover, cultural influences may moderate this relationship, since happiness perception is tightly linked to culture as mentioned earlier. Prior research suggests that some individuals, particularly those with early adverse experiences, may have been conditioned to associate happiness with punishment or guilt, leading them to avoid positive emotions altogether [19]. Moreover, the belief that happiness is fleeting can lead to pessimism, lower optimism, and increased vulnerability to depression, anxiety, and stress [19]. This psychological pattern may be especially relevant in societies experiencing chronic instability, such as Lebanon, where unpredictable economic, political, and social stressors heighten uncertainty about the future.

The mean value of the total FHS can be considered high in our sample (13.33), being higher than what was found in previous studies [12,21–23]. While this finding should be interpreted cautiously, it may reflect the unique context of Lebanon where several cultural factors can lead to the fear of happiness, like religious beliefs, superstitious beliefs, add to this the multiple stressors that affected Lebanon. Given that Lebanon has experienced severe financial collapse and political instability, it is plausible that these stressors contribute to a greater fear of happiness, reinforcing the belief that joy is short-lived or can lead to misfortune. This finding underscores the need for further research to explore cultural, historical, and psychological factors contributing to fear of happiness in Arabic-speaking populations.

### Clinical implications

The findings of the present study have important implications for both clinical practice and psychological research within the Arabic-speaking populations. Beyond its descriptive findings, the primary contribution of this study lies in the validation of the Arabic version of the FHS. The availability of a brief, reliable and culturally validated measure enables clinicians and researchers to assess beliefs related to happiness using an instrument that is linguistically and conceptually appropriate.

From a clinical perspective, fear of happiness represents a cognitive and emotional belief system that may influence emotional regulation strategies, engagement with positive experiences, and responsiveness to therapeutic interventions [11,19]. Individuals who hold beliefs that happiness is dangerous, morally questionable or likely to be followed by negative consequences may consciously or unconsciously suppress positive emotions. As a result, fear of happiness may function as a psychological barrier within treatment, particularly in interventions that explicitly aim to increase positive affect. This has direct relevance for treatment of depression and anxiety.

Many evidence-based therapeutic approaches, including behavioral activation, pleasant activities scheduling, gratitude-based exercises and other positive-oriented interventions, rely on increasing engagement with rewarding experiences and cultivating positive emotional states [58–60]. However, research indicates that some individuals actively avoid positive emotions or experience them as threatening, particularly in the context of depression and emotion regulation difficulties [61,62]. For individuals with elevated fear of happiness, such interventions may be experienced as aversive or ineffective. If fear of happiness is not identified and addressed, clients may resist, disengage from positive-focused components of therapy, potentially limiting treatment response. Assessing fear of happiness may therefore help clinicians anticipate barriers to interventions, refine case formulation and adapt therapeutic strategies in ways that are more psychologically and culturally acceptable to the individual.

Importantly, fear of happiness should not be conceptualized only as an individual vulnerability, but also as a cultural belief system. In Arabic-speaking contexts, religious values, collectivist norms, moral beliefs and prolonged exposure to sociopolitical stressors may shape attitudes toward emotional expression of happiness [2,11]. The Arabic FHS provides clinicians with a culturally sensitive tool to explore these beliefs without pathologizing culturally normative attitudes, thereby supporting nuanced clinical formulation.

From a research perspective, the Arabic FHS facilitates the inclusion of Arabic-speaking populations in cross-cultural studies of well-being and positive psychology, contributing to greater cultural equity in psychological assessment. The scale may be particularly useful in examining how fear of happiness interacts with constructs such as trauma exposure, emotion regulation strategies, religiosity, coping under chronic stress and treatment engagement. Future research should extend the present findings through longitudinal and intervention studies to clarify how fear of happiness influences therapeutic processes and outcomes and whether targeting maladaptive beliefs about happiness enhances response to treatment.

### Study limitations

This study has several limitations that should be acknowledged. First, the use of a snowball sampling method may limit generalizability of the findings. Due to the nature of this recruitment strategy, it was not possible to calculate a response rate, which limits conclusions regarding representativeness of the sample. Although the online survey was configured to allow only one response per Google account, thereby reducing the likelihood of duplicate submissions, it remains theoretically possible that some participants could have responded more than once using different accounts. Second, participants were primarily educated individuals with internet access, which may not fully represent broader Arabic-speaking populations. The sample included a broad age range, and findings may vary across different age groups. Women were overrepresented, which may further affect generalizability. Future studies should utilize randomized or stratified sampling to enhance generalizability. In addition, the sample being from the Lebanese population, we cannot generalize the results on all Arabic-speaking countries and further research needs to be done to address cultural differences within the Arabic countries. An additional methodological consideration is the elevated RMSEA observed in the CFA despite excellent CFI and SRMR values. Therefore, the factorial validity of the Arabic FHS should be interpreted in conjunction with multiple fit indices rather than relying solely on the RMSEA. Third, the study relied on self-reported online surveys, which may introduce response biases and social desirability effects. The response time was not recorded or used as an indicator of response attention. Finally, consistent with recommendations that psychometric validation is an iterative process, several important properties were not assessed in the present study, including test-retest reliability, predictive validity and inter-rater reliability.

## Conclusion

The current study evaluated the psychometric properties of the Arabic version of the FHS in a sample of Lebanese adults. The findings represent a foundational step in the cross-cultural validation of the FHS, providing a basis for further psychometric refinement and application. By making a brief and culturally validated Arabic version of the FHS available, this study contributes to ongoing efforts to improve culturally sensitive assessment within positive psychology. This scale is a major addition toward more elaborate research in Lebanon and Arabic-speaking countries in various topics addressing wellbeing, emotion regulation and beliefs about happiness under conditions of social and economic adversity. It is hoped that the availability of this instrument will stimulate research in this field within Arab countries. Future studies should further examine the factorial structure of the Arabic FHS in larger and more diverse Arab populations. Replication using independent samples may help confirm the stability of the one-factor structure and further clarify the observed discrepancy between the RMSEA and the other model fit indices.

## Supporting information

S1 File Fear of happiness scale in English. (PDF)
