# Supplementary material for: Psychometric properties of the Arabic Fear of Happiness Scale in a sample of Lebanese adults
Source: PLoS One. 2026 Sep 15;21(9):e0356389. doi: 10.1371/journal.pone.0356389 (PMC13577388; doi:10.1371/journal.pone.0356389)
Supplement: Supplementary file 1 [file pone.0356389.s001.pdf]

Fear of Happiness Scale

Please rate the extent to which you agree with each statement, using the scale from 1 to 7 as shown below.

1	2	3	4	5	6	7
Strongly disagree	Somewhat disagree	A little disagree	Neither Agree or Disagree	A little agree	Somewhat agree	Strongly agree

1. I prefer not to be too joyful, because usually joy is followed by sadness.	1	2	3	4	5	6	7
2. I believe the more cheerful and happy I am, the more I should expect bad things to occur in my life.	1	2	3	4	5	6	7
3. Disasters often follow good fortune.	1	2	3	4	5	6	7
4. Having lots of joy and fun causes bad things to happen.	1	2	3	4	5	6	7
5. Excessive joy has some bad consequences.	1	2	3	4	5	6	7

References

Joshanloo, M. (2013). The influence of fear of happiness beliefs on responses to the satisfaction with life scale. *Personality and Individual Differences*, *54*(5), 647-651. https://doi.org/10.1016/j.paid.2012.11.011

Joshanloo, M., Lepshokova, Z. Kh., Panyusheva, T., Natalia, A. Poon, W.C., Yeung, V.W., Sundaram, S., Achoui, M., Asano, R., Igarashi, T., Tsukamoto, S., Rizwan, M., Khilji, I., A., Ferreira, M.C., Pang, J.S., Ho, L.S., Han, G., Bae, J., & Jiang, D. (2014). Cross-cultural validation of the fear of happiness scale across 14 national groups. *Journal of cross-cultural psychology*, *45*(2), 246-264. https://doi.org/10.1177/0022022113505357
